# Anti-TGF-β attenuates tumor growth via polarization of tumor associated neutrophils towards an anti-tumor phenotype in colorectal cancer

**DOI:** 10.7150/jca.38179

**Published:** 2020-02-14

**Authors:** Fengxian Qin, Xiaoyong Liu, Jifei Chen, Shishun Huang, Wei Wei, Yan Zou, Xuexiang Liu, Kaifeng Deng, Shanying Mo, Jianming Chen, Xiaoli Chen, Yujie Huang, Weijun Liang

**Affiliations:** 1 Medical Science Laboratory, the Fourth Affiliated Hospital of Guangxi Medical University, Liuzhou, Guangxi, P. R. China 545005; 2 Department of Clinical Laboratory, Liuzhou Municipal Liutie Center Hospital, Liuzhou, Guangxi, P. R. China 545007

**Keywords:** tumor associated neutrophils, colorectal cancer, Anti-TGF-β, polarization, phenotype

## Abstract

Tumor associated neutrophils (TANs) play important roles in the progress of CRC. Since tumor microenvironments could influence the phenotypes of TANs, altering the tumor microenvironment to polarize the phenotype of TANs may be a new strategy for tumor treatment. This study aims to investigate the effect of anti-TGF-β on the polarization of TANs from a pro-tumor phenotype towards an anti-tumor phenotype in CRC. In this work, CRC patients had more infiltration of TANs and higher expression of TGF-β in CRC tissue when compared with the controls. *In vitro*, SW480 cells were co-cultured with primed neutrophils, which simulated the TANs in the tumor microenvironment, and TGF-β was blocked by anti-TGF-β (1D11) in order to polarize TANs. Anti-TGF-β treatment increased the cytotoxicity of TANs and decreased the metastatic chemoattractants secreted by TANs, and ultimately increased the apoptosis of CRC cells significantly while remarkably suppressing the migration of tumor cells. The changes of signaling pathways in the TANs and tumor cells were explored. The results showed that anti-TGF-β attenuated CRC may be partly mediated by suppression of PI3K/AKT signaling pathways in TANs and partly mediated by suppression of TGF-β/Smad signaling pathways in tumor cells. Furthermore, the tumor in the mice treated with 1D11 was obviously smaller and had reverse tumorigenesis compared with the controls, while neutrophil depletion reduced the anti-tumor effect of 1D11. Our data suggest that anti-TGF-β attenuates tumor growth via the polarization of TANs to an anti-tumor phenotype in CRC, which provides new strategies for CRC treatment.

## Introduction

Colorectal cancer (CRC) is a common cancer in the world and the third leading cause of cancer mortality in United States 1. The pathogenesis of CRC is complex, but chronic inflammation accounts for about 25% of all CRC patients 2. It indicates the importance of chronic inflammation in colon cancer. Inflammation participates in the progression of many malignancies 3. Tumor cells produce various inflammatory cytokines. These cytokines recruit inflammatory cells such as neutrophils to the tumor site and activate them to promote tumor growth and progression 4. A mass of neutrophils have been found in the lamina propria and submucosa of ulcerative colitis (UC) and ultimately progress to UC-associated colon carcinogenesis 5. High level of neutrophil-to-lymphocyte ratio (NLR) and a large number of infiltrated neutrophils in tumor tissue show a poor prognosis in a variety of tumor 6-9. Numerous clinical data also confirm the bad prognostic effects of neutrophils in other tumors 10. Additionally, the neutrophils infiltrated in the tumor tissues, namely tumor-associated neutrophils (TANs) are not only positively correlated with clinical stage, but also act as an independent prognostic factor in multivariate analysis in patients with CRC 11. This indicated that TANs may be a potential treatment target for CRC.

Neutrophils, also known as polymorphonuclear leukocytes (PMN), play an important role in defending human hosts from bacterial infection. The neutrophils show different phenotypes with diverse functional programs under the special conditions. The tumor microenvironment forms a special niche that influences the phenotype characteristics of infiltration cells 12. Same as the phenotypic macrophages (M1/M2), the concept of immune cell polarization also has been extended on TANs 13. N1 neutrophils show an anti-tumor phenotype with their capability of efficiently killing microorganisms and tumor cells, while N2 neutrophils show a pro-tumor phenotype 14 with synthesizing numerous cytokines, such as matrix metalloproteinase 2/9 (MMP-2/9), arginase 1 (arg-1), neutrophil elastase (NE), vascular endothelial growth factor (VEGF) and so on 15. These cytokines promote tumor angiogenesis, tumor cell dissemination, and tumor cell metastasis 16-18. Researches have proposed that TANs can be phenotypic or functional plastic, which are modulated by cues presented in the tumor environment 19. Since the tumor microenvironment influences the phenotype of TANs, alteration of the tumor microenvironment may polarize the phenotype of TANs. Andzinski et al 20 had confirmed that type I IFNs could alter the pro-tumor phenotype of neutrophils into anti-tumor phenotype in their recent study. Therefore, alteration of neutrophil phenotypes may provide a potential therapeutic strategy in cancer treatment. But modulation of neutrophil phenotypes in CRC is still poorly defined.

Adverse prognostic effects of high expression on transforming growth factor β (TGF-β) have been confirmed in a variety of tumors 21,22. Emerging evidence suggests high expression of TGF-β could promote tumor cell migration, invasion, and survival 21-23, and TGF-β signaling inhibition is an emerging strategy for cancer therapy. High level of TGF-β is also found in CRC and plays a promoting tumor role in the process of CRC 24. It is worth mentioning, recent works showed that TANs could be modulated and polarized towards an N1 phenotype by systemic inhibition of TGF-β using a specific Alk5 kinase inhibitor called SM16 in mice. This raises the hypothesis that suppression on the level of TGF-β in CRC may, on one hand, inhibit its own signal pathway, and on the other hand, polarize the TANs to an anti-tumor phenotype, which ultimately attenuates tumor growth.

Whether inhibition of TGF-β in the tumor microenvironment could polarize the pro-tumor TANs to an anti-tumor phenotype and ultimately attenuate tumor growth in CRC? To answer this question, the anti-TGF-β (1D11) was used to suppress the level of TGF-β in the co-culture system of CRC cells and TANs *in vitro* and to inhibit TGF-β expression in mice tumor model. Our works showed that TGF-β blocking could significantly increase the apoptosis of CRC cells while suppressing the migration of tumor cells remarkably. Furthermore, the mice treated with 1D11 showed markedly smaller size of tumors and reversed tumorigenesis compared with the controls, even compared with the 1D11 treated mice that had TANs depletion with neutrophil-neutralizing anti-Ly6G simultaneously. The correlated mechanism may be partly mediated by suppression of PI3K/AKT signaling pathways in TANs and partly by suppression of TGF-β/Smad signaling pathways in tumor cells. Our data indicate that anti-TGF-β could polarize TANs to an anti-tumor phenotype and then attenuate tumor growth in CRC. In addition, our work also suggests that anti-TGF-β attenuates tumor growth is dependent on a synergistic combination of effects on both the tumor cells and TANs in microenvironment, which provides new strategies for CRC treatment.

## Materials and Methods

### Patients and specimens

Patients who were pathologically diagnosed with CRC at the Fourth Affiliated Hospital of Guangxi Medical University from January 2016 to December 2018 were enrolled in this study. Patients were excluded if 1) they had other inflammatory disease or any clinical evidence of infection; 2) they had sporadic colorectal cancer or combined with other tumors; 3) they had received preoperative chemotherapy before; 4) they failed in clinical follow-up. Finally, 108 patients were included in this study. A portion of their tumors and adjacent normal tissues were collected for research before they received any anti-cancer therapy. Moreover, the blood of all patients was also collected. Stages of tumor were classified with Dukes stage criteria. Baseline clinical data of the participants were summarized in Table 1. One hundred and ten healthy human volunteers were set as controls. All participants signed on the informed consents. This protocol was approved by the Research Ethics Committee at the Fourth Affiliated Hospital of Guangxi Medical University.

### Colorectal cancer cell conditioned medium

SW480 cells (a human colorectal cancer cell line) were plated in DMEM medium (GIBCO, NY, USA) containing 10% complement-free fetal bovine serum (FBS, GIBCO, NY, USA) with a mixture of antibiotics (streptomycin and penicillin), and maintained at 37 ℃ with 5% CO_2_. When cells were 50% confluent, the medium in the plate was replaced by the DMEM medium supplemented with 1% FBS. The conditioned medium was collected 48 hours later and further used to prime neutrophils.

### Purification of neutrophils

Heparin-anticoagulated blood (5-10 mL) was obtained from healthy human volunteers. All volunteers were provided with written informed consents. The neutrophils in the blood were isolated by density centrifugation using Polymorphprep (Nycomed, Oslo, Norway) following with the manufacturer's instructions. Trypan blue exclusion (Sigma Aldrich, MO, USA) was used to check the viability of cells. The purity of cells was checked by Wright staining of cytocentrifuge slides. The purity of the granulocytes was over 95%. Those neutrophils were used for further culture.

### Neutrophil priming

The neutrophils were cultured with the conditioned medium of SW480 cells for six hours for priming before being co-cultured with SW480 to adjust to the environment in cancer cell lines. In this way, the processed neutrophils can be considered as TANs before being co-cultured with colorectal cancer cells.

### Colorectal cancer cells and primed neutrophils co-culture

SW480 cells and primed neutrophils were co-cultured under the condition with transwell inserts (0.4µm pore, Corning Coster Corp. MA, USA) and six-well plastic culture plates (Corning Coster Corp. MA, USA). Briefly, primed neutrophils were counted and plated at an appropriate concentration with SW480 cells in DMEM medium supplemented with 10% FBS. Furthermore, direct co-culture of SW480 cells and primed neutrophils were also performed to explore whether they interact with each other via a contact-dependent mechanism.

### Assessment of neutrophil to lymphocyte ratio (NLR)

The complete blood counts (CBC) with automated differential counts were performed to detect the neutrophil and lymphocyte counts in all blood samples that were taken on admission for initial diagnosis. The NLR was calculated through dividing the number of neutrophils by the number of lymphocytes.

### Cell cycle and cell apoptosis analysis

Tumor cells were grown (5×10^5^) on six-well plates, and then co-cultured with or without primed neutrophils for 12 hours in the medium in presence or absence of 1D11 (R&D Systems, Inc., MN, USA). For cell cycle analysis, the cells were stained with Propidium Iodide (PI) and detected by flow cytometry. For cell apoptosis analysis, the cells were stained with Annexin V-FITC/PI Apoptosis Detection kit (KeyGen Biotech Co., Nanjing, China) according to the manufacturer's instructions, and the apoptosis of cells were analyzed by flow cytometry.

### Wound migration assay

When SW480 cells reached 80% confluent in six- well plates, the monolayer of cells were wounded with P200 pipette tips and then co-cultured with/ without primed neutrophils in the medium in presence or absence of 1D11. Twelve hours later, the wound area in each group was measured under microscope.

### Enzyme-linked immunosorbent assay (ELISA)

The protein levels of MMP9, TGF-β, TNF-α, arg-1, NE, IL-6, GM-CSF and IFN-γ were detected using the corresponding ELISA kits (purchased from Ray Biotech, GA, USA) following the protocols supplied by the manufacturers. To explore the protein expression in specific cells, SW480 and primed neutrophils were co-cultured for 12 hours and then separately cultured in DMEM and RPMI 1640 (GIBCO, NY, USA) medium containing 1% FBS for another 6 hours. Cellular supernatants were collected for protein detection.

### Quantification of intracellular reactive oxygen species (ROS) generation in primed neutrophils

Primed neutrophils and SW480 cells were co-cultured in the six-well plates at an appropriate concentration for 12 hours with or without 1D11. To determine the intracellular ROS generation in primed neutrophils, Dichlorodihydrofluorescein diacetate (DCFH-DA, Invitrogen, CA, USA) assay was used as previously described 25,26. Briefly, neutrophils were collected from the co-cultured system and incubated in the culture media supplemented with 3 mM DCFH-DA at 37℃. The cells were washed with PBS 30 minutes later, and flow cytometry was used to detect the DCFH-DA fluorescence on the cells (Beckman Coulter, Inc., FL, USA).

### Secretion of NO and H_2_O_2_ by priming neutrophils

The media of co-culture system were collected, and the production of NO was determined with the protocol of Griess reagent system kit (Promega, WI, USA). H_2_O_2_ levels were evaluated using a Hydrogen Peroxide Colorimetric Detection Kit (Abnova, Taiwan, China).

### Histopathological and immunohistochemical staining

Four-micrometer sections of paraffin-embedded tumor tissue samples were cut and stained with hematoxylin and eosin. For immunohistochemical staining, the slices were treated with 3% hydrogen peroxide to block the endogenous peroxidase activity, and further incubated with 0.1 M citrate buffer for 5 minutes to enhance immunoreactivity. After that, the sections were incubated with anti-TGF-β (Abcam, London, UK) and anti-ki67 (Abcam, London, UK) overnight at 4℃. Goat anti-rabbit IgG (Zsbio Commerce Store, Beijing, China) was used as secondary antibody. The slides were scanned under digital microscope.

### Quantitative real time PCR (qPCR)

We performed qPCR using SYBR Green PCR Mix Kit (Takara, Dalian, China) on Bio-rad ABI 7500 PCR system (Bio-Rad, Berkeley, CA, USA). Each targeted and standard GAPDH cDNA was made in triplicates. The expression of target gene was analyzed with the comparative Ct method and the relative quantification of gene expression was normalized to GAPDH. The primer sequences were shown in Table 2.

### FCM analysis

To prepare single-cell suspensions, the tumor tissues were minced and then incubated in the digestion buffer (RPMI 1640, 5% FBS, 1 mg/ml collagenase, 30 μg/ml DNase) at 37 °C for 30 minutes. To determine the infiltration of TANs in tumor tissue, single-cell suspensions were stained with CD66-FITC (eBioscience, CA, USA) and CD15-PE (eBioscience, CA, USA) and then sorted by flow cytometry.

### Western blot analysis

The proteins were extracted as previously described 27,28. The cells in each group were lysed with cell lysis buffer supplemented with PhosSTOP phosphatase inhibitor cocktails (Roche, IN, USA). Thirty minutes later, the cell lysates were centrifuged at 12,000 g at 4 °C for 30 minutes, and then the supernatants were collected. Concentration of protein was performed using the bicinchonininc acid protein assay reagent kit. Same amount of proteins (50 µg) in each group were subjected to 10% SDS-PAGE and transferred to PVDF membranes. After blocking with 5% skim milk for 2 hours, the membranes were incubated with anti-PI3K (eBioscience, CA, USA, 1:1,000), anti-AKT (eBioscience, CA, USA, 1:1,000), anti-p-AKT (eBioscience, CA, USA, 1:1,000), anti-TGF-β (eBioscience, CA, USA, 1:1,000), anti-SMAD7 (eBioscience, CA, USA, 1:1,000) or anti-β-actin (eBioscience, CA, USA, 1:1,000). Twenty-four hours later, the membranes were washed and incubated with secondary antibody, and then detected with the enhanced chemiluminescence regents one hour later (Bio-Rad, CA, USA).

### Xenograft model

Forty athymic nude mice were purchased from the Model Animal Research Center at Guangxi Medical University, and housed under pathogen-free facility. Each mouse was conducted with an inguinal subcutaneous injection of 2×10^6^ SW480 cells as described in previous study 29. The mice were randomly divided into four groups: PBS (xenograft mice only with PBS treatment), anti-PMN (PMN in the mice were eliminated with anti-Ly6G, three times per week, i.p, 5 mg/Kg), 1D11 (1D11 was used to inhibit the TGF-β expression in the mice, three times per week, i.p, 5 mg/Kg), and anti-PMN+1D11 (the mice were treated with anti-Ly6G and 1D11 simultaneously). The treatments were performed one day later after cell inoculation. Tumor growth and mortality, and the body weights of mice were calculated every two days. The experiment was approved by the Animal Ethics Committee at the Fourth Affiliated Hospital of Guangxi Medical University.

### Azoxymethane (AOM) and dextran sulfate sodium (DSS) induced CRC Mouse Model

Fifty female C57BL/6 mice of 7 weeks old purchased from the SLAC Laboratory Animal Central (Changsha, Hunan, China) were maintained under standard laboratory conditions. After acclimating for a week, the mice were randomly assigned into one of five groups. The grouping methods used in the AOM/DSS model were same as those in xenograft model. In addition, the mice treated with ddH_2_O and PBS were set as negative control. Briefly, except for the negative control group, all the mice were intraperitoneally injected with AOM (10 mg/kg, Sigma-Aldrich, MO, USA). Seven days later, 2% DSS was administered to mice in drinking water for 7 days. In the later 14 days, the mice were allowed to access untreated water *ad libitum*. Seven days of 2% DSS and 14 days of untreated water constituted 1 cycle, and three cycles were employed in this experiment. Mice were monitored two times weekly for body weights. In some experiments, 1D11 and/or anti-Ly6G were injected into the tail veins of mice three times per week at a ratio of 5 mg/Kg, starting one day after DSS treatment. At the end of the third cycle, the mice were sacrificed and the colons were removed and measured. All experiments were carried out following protocols approved by the Animal Ethics Committee at the Fourth Affiliated Hospital of Guangxi Medical University.

### Statistical analyses

Data from experiments are presented as the means ± SEM. One-way ANOVA test or Student's t test was used to evaluate the statistical differences with GraphPad Prism (version 5.0) statistical program. *P*<0.05 was considered significant.

## Results

### The TANs infiltration, TGF-β, and NLR expression in CRC patients

**Figure 1:**
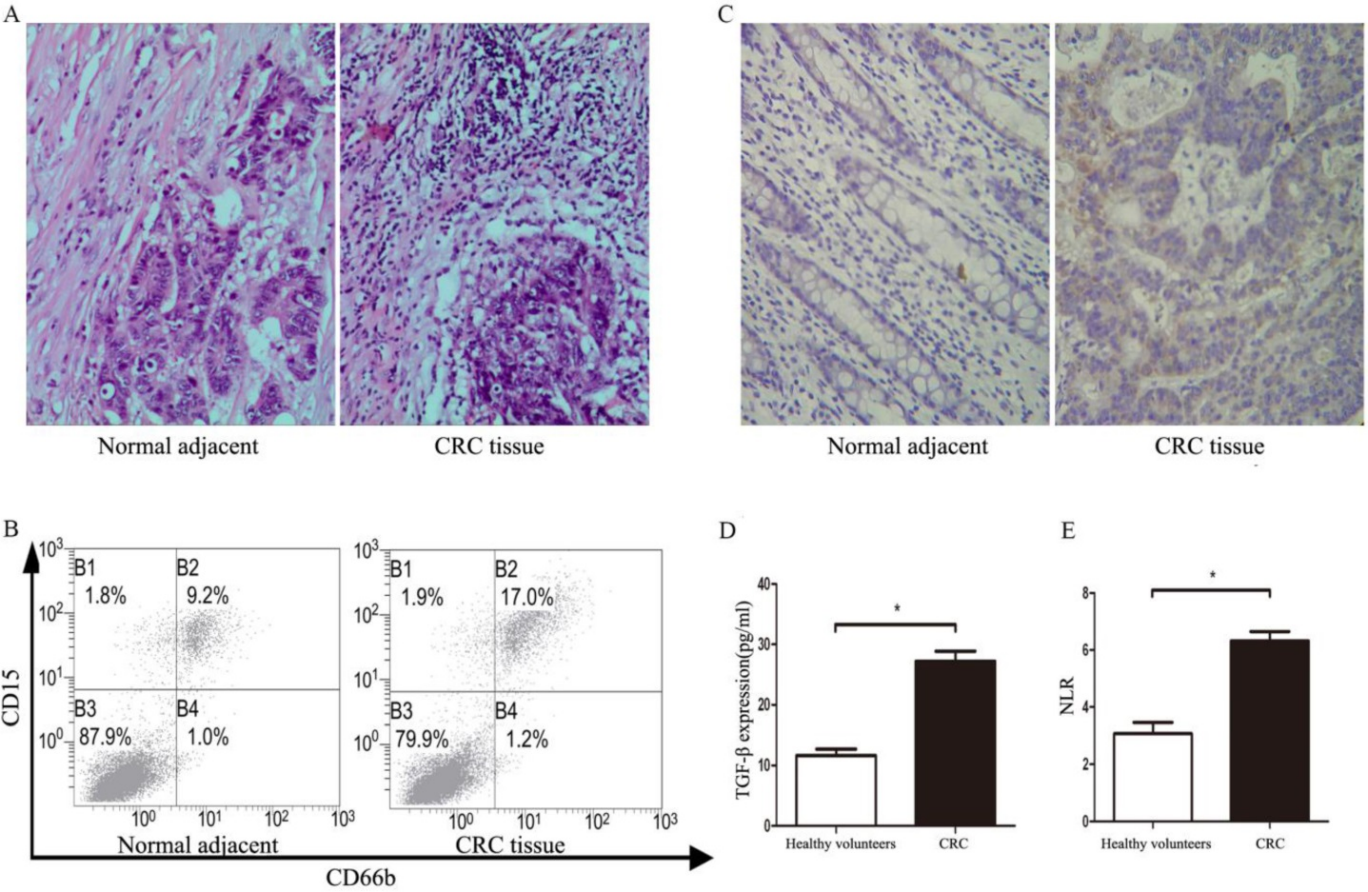
** The TANs infiltration, TGF-β, and NLR expression statues in CRC patients.** A. The infiltration of TANs in CRC tumor tissues and corresponding adjacent normal tissues detected by HE. B. The infiltration of TANs in CRC tumor tissues and corresponding adjacent normal tissues detected by flow cytometry. C. The expression of TGF-β in CRC tumor tissues and corresponding adjacent normal tissues detected by flow immunohistochemical staining. D. The expression of TGF-β in the serum of CRC and healthy controls detected by ELISA. E. The level of NLR in the peripheral blood of CRC patients and healthy controls. Asterisks (*) indicated significant differences between healthy volunteers and CRC patients.

A total of 108 patients were recruited from April 2016 to December 2018. Baseline characteristics were summarized in Table 1. The infiltration of TANs and expression of TGF-β were detected in tumor tissues. As shown in Figure 1A, the infiltration of TANs were increased remarkably when compared with corresponding adjacent normal tissues (*P*<0.05). The results of flow cytometry also showed the infiltration of TANs were significantly elevated in primary tumor tissues when compared with the normal controls (Figure 1B, *P*<0.05). Similarly, the level of TGF-β in primary tumor tissues was significantly increased when compared with the adjacent normal tissues (Figure 1C, *P*<0.05), and a similar result was confirmed in the serum of CRC patients (Figure 1D, *P*<0.05). All data on neutrophil counts and lymphocyte counts from patients were collected, so that the NLR could be calculated for all patients. Compared with the healthy volunteers, the NLR was significantly increased in CRC patients (Figure 1E, *P*<0.05).

### Inhibition of TGF-β signaling promoted apoptosis and suppressed migration in CRC cells

**Figure 2:**
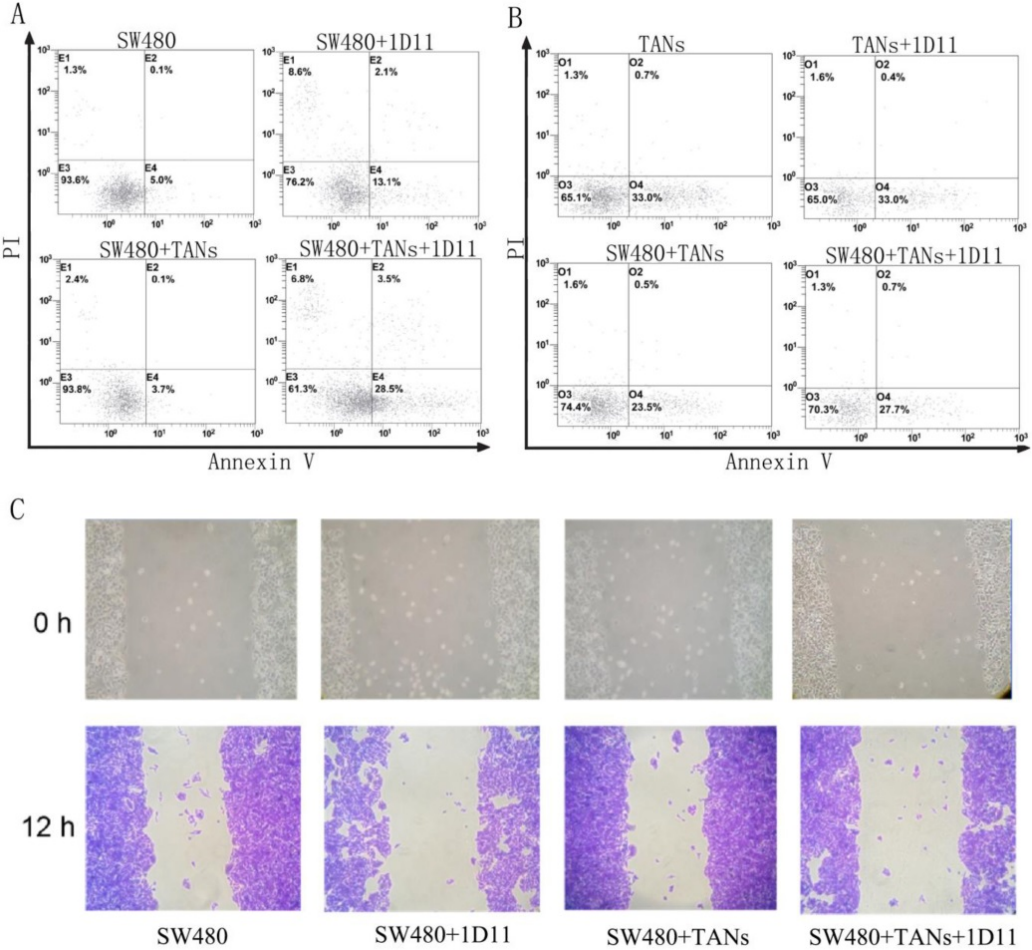
** TGF-β blockade promoted apoptosis and suppressed migration of tumor cells.** SW480 cells were co-cultured with TANs at a ratio of 1:2, and 1D11 was used to inhibit the expression of TGF-β in the culture system (2 µg/ml, 8 hours). The apoptosis of cells were detected by flow cytometry, and the migration of tumor cells were assessed by wound migration assay. A. Effect of 1D11 on the apoptosis of SW480 cells in each group. B. Effect of 1D11 on the apoptosis of TANs in each group. C. Effect of 1D11 on the migration of SW480 cells in each group.

To evaluate the function of neutrophils treated with 1D11, the cytotoxicity of neutrophils against tumor cells was examined. SW480 cells were co-cultured with primed neutrophils (equivalent to TANs) at a ratio of 1:2. Eight hours later, the surviving tumor cells were measured. As shown in Figure 2A, in direct co-culture of SW480 and TANs system, but not the transwell co-culture system (data not shown), a highest apoptosis of SW480 cells were shown when treated with 1D11 (*P*<0.05). It is worth mentioning that the apoptosis of TANs were also detected, and a lowest apoptosis of TANs were shown in the co-culture system when compared with other groups. After 1D11 treatment, the apoptosis of TANs appeared to rise again (Figure 2B, *P*<0.05). The cycle of tumor cells in each group was detected, while there was no significance (data not shown). Furthermore, the migrations of tumor cells were investigated. As shown in Figure 2C, inhibition of TGF-β signaling caused a prominent decrease of tumor migration in the direct co-cultured system when treated with 1D11 (*P*<0.05). All above suggested that inhibition of TGF-β signaling could up-regulate the apoptosis and down-regulate the migration potential of malignant cells in the co-culture system via a contact-dependent mechanism.

### TGF-β blockade promoted tumor cell apoptosis via increasing the cytotoxicity potential of TANs

**Figure 3:**
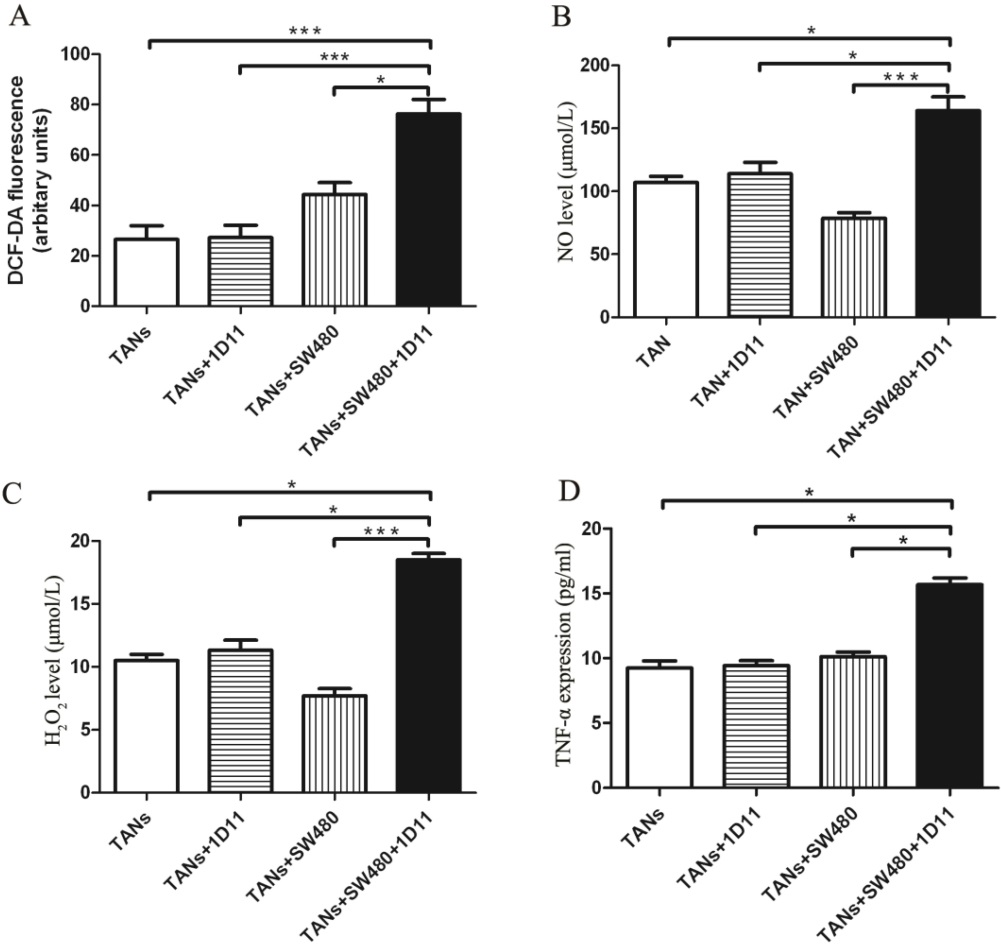
** TGF-β blockade increased the cytotoxicity potential of TANs.** TANs were co-cultured with SW480 cells at a ratio of 2:1, and 1D11 was used to inhibit the expression of TGF-β secreted by tumor cells (2 µg/ml, 12 hours). The cytotoxicity potential of TANs was detected in each group. For TNF-α detection, the TANs in each group were isolated from the co-cultured system and maintained in the medium containing 1% FBS for another 6 hours, and the cellular supernatants were collected to detect TNF-α expression. The expressions of ROS (A), NO (B), H_2_O_2_ (C), and TNF-α (D) in TANs in the culture system with/without 1D11 treatment were demonstrated. Asterisks (*) indicated significant differences in relation to TANs, TANs+1D11, or TANs+SW480 group cells.

Whether TGF-β inhibition has any effect on the TANs' cytotoxicity against tumor cells? The cytotoxicity potential of TANs was analyzed. The expressions of neutrophil cytotoxic mediators including ROS, NO, H_2_O_2_, and TNF-α were detected in each group. Interestingly, the co-culture system with 1D11 treating group showed increased ROS production when compared with other controls (Figure 3A, *P*<0.05). Similarly, the co-culture system with 1D11 treating group showed highest expression of NO and H_2_O_2_ when compared with others (Figure 3B and Figure 3C, *P*<0.05). TNF-α may promote or inhibit the progression of tumor depending on its concentration 30,31. One significant feature of N1 TANs is the TNF-α production at tumor sites 32. To evaluate the effect of 1D11 on production of TNF-α in TANs, the level of TNF-α in each group was assessed. A highest expression of TNF-α could be found in the co-culture system with 1D11 treating group when compared with other controls (Figure 3D, *P*<0.05).

### TGF-β blockade inhibited tumor cell migration via decreasing the metastasis chemoattractants secreted by TANs

**Figure 4:**
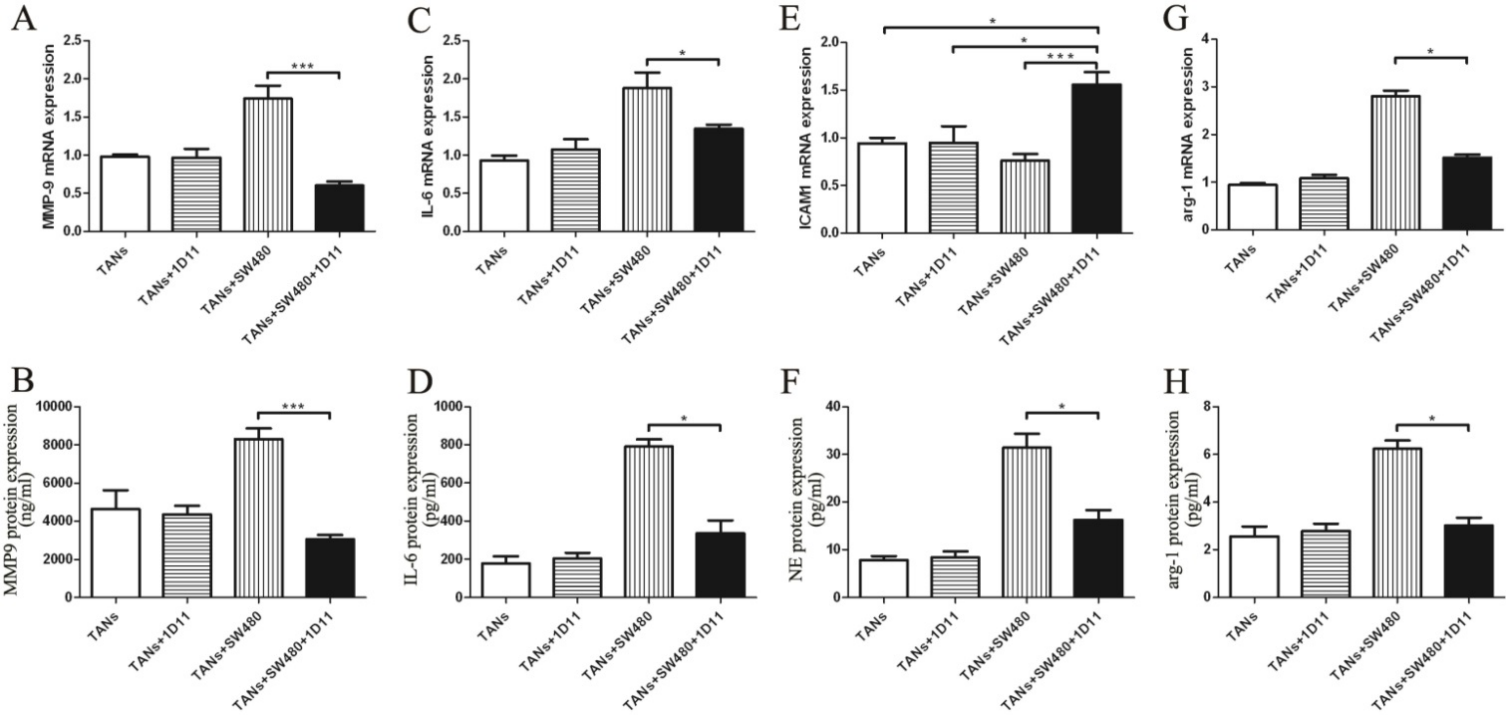
** TGF-β blockade decreased the metastasis chemoattractants secreted by TANs.** TANs were co-cultured with SW480 cells at a ratio of 2:1, and 1D11 was used to inhibit the expression of TGF-β secreted by tumor cells (2 µg/ml). Twelve hours later, the TANs in each group were isolated from the co-cultured system and maintained in the medium containing 1% FBS for another 6 hours, and the cellular supernatants were collected to detect the protein expression of metastasis chemoattractants by ELISA, while the cells were collected to detect the mRNA expression of metastasis chemoattractants by qPCR. The mRNA levels of *MMP9* (A), *IL-6* (C), *ICAM-1* (E), and *arg-1* (G) in TANs in the culture system with/without 1D11 treatment were demonstrated. The protein levels of MMP9 (B), IL-6 (D), NE (F), and arg-1 (H) in TANs in the culture system with/without 1D11 treatment were demonstrated. Asterisks (*) indicated significant differences in relation to TANs, TANs+1D11, or TANs+SW480 group.

To evaluate the level change of cytokine in tumor microenvironment after TGF-β blocking, we examined the metastasis chemoattractants secreted by primed neutrophils in the microenvironment, including MMP-9, IL-6, ICAM-1, and NE. *MMP-9* mRNA expression was remarkably reduced in the co-cultured system treated with 1D11 when compared to that without 1D11 treatment, so did the protein expression (Figure 4A and Figure 4B, *P*<0.05). Similarly, a lower expression of IL-6 (mRNA and protein) was shown in the co-cultured system treated with 1D11 when compared with that without 1D11 treatment (Figure 4C and Figure 4D, *P*<0.05). The adhesion molecule ICAM-1 is associated with neutrophil maturation and activation 33. The result showed that mRNA expression of *ICAM1* on the primed neutrophils was significantly up-regulated in the co-culture system treated with 1D11 when compared with others (Figure 4E, *P*<0.05). NE, a neutrophil-derived proteinase, plays an important role in promoting tumor cell proliferation. As shown in Figure 4F, the level of NE was remarkably decreased in the co-culture system treated with 1D11 when compared with that without 1D11 treatment (*P*<0.05). In order to explore whether TGF-β blockade has some effects on the expressions of immunosuppressive molecules in TANs, the expression of immunosuppressive molecules arg-1 was detected in each group. As shown in Figure 4G, the mRNA level of *arg-1* in the co-culture system treated with 1D11 was 2- to 3-fold lower when compared with the co-cultured group without 1D11 treatment (*P*<0.05). There was a same trend for the protein expression of arg-1 (Figure 4H, *P*<0.05).

### TGF-β blockade improved GM-CSF and INF-γ expression in tumor microenvironment

**Figure 5:**
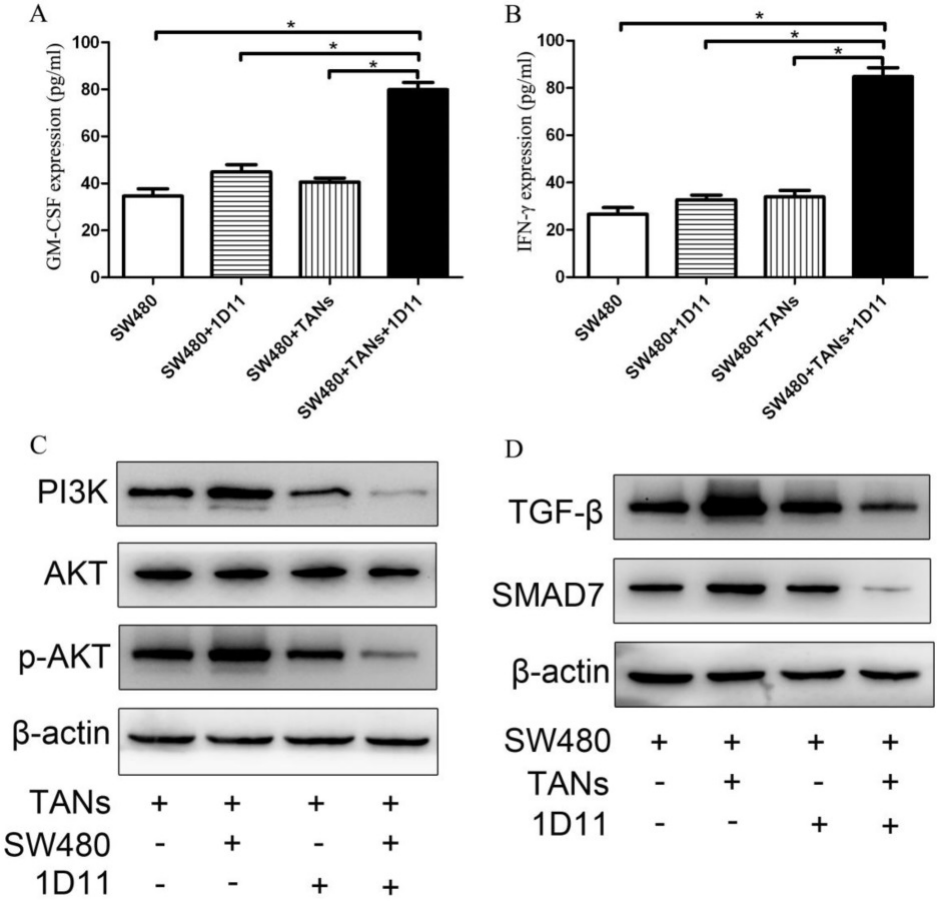
** Effect of 1D11 on the expressions of GM-CSF and IFN-γ in tumor cells and on the signaling pathways change in the co-culture system.** TANs were co-cultured with SW480 cells at a ratio of 2:1, and 1D11 was used to inhibit the expression of TGF-β secreted by tumor cells (2 µg/ml). SW480 and primed neutrophils were co-cultured for 12 hours and then separately cultured in medium containing 1% FBS for another 6 hours, respectively. Cellular supernatants were collected to detect the GM-CSF and IFN-γ expressions in tumor cells by ELISA. The signaling pathway changes in the cells were detected by Western Blot. (A, B). The expressions of GM-CSF and IFN-γ in SW480 in the culture system with/without 1D11 treatment were demonstrated. C. The protein expressions of PI3K, AKT, and p-AKT in TANs in the culture system with/without 1D11 treatment were demonstrated. D. The protein expressions of TGF-β and SMAD7 in SW480 in the culture system with/without 1D11 treatment were demonstrated. Asterisks (*) indicated significant differences in relation to SW480, SW480+1D11, or SW480+TANs group.

Since the differentiation of neutrophils is regulated by multiple cytokines such as G-CSF, GM-CSF, and hematopoietic growth factors, while these factors may be also produced by tumors. To explore whether TGF-β blockade has any effect on the tumor cells which influences the phenotype of TANs in the co-culture system, the expressions of GM-CSF and INF-γ in SW480 cells were detected. As shown in Figure 5, the protein expressions of GM-CSF and IFN-γ were significantly increased respectively in the co-culture system treated with 1D11 when compared with other groups (*P*<0.05).

### TGF-β blockade suppressed the activation of the PI3K/AKT pathway in TANs and TGF-β/Smad signaling pathway in tumor cells

PI3K/Akt has been implicated to play an important role in regulating the chemotaxis of neutrophil 34. To investigate whether PI3K/Akt is involved in the polarization of TANs, we used immunoblotting to analyze the PI3K/Akt activity in 1D11 treated TANs. As shown in Figure 5C, 1D11 treatment suppressed the activation of the PI3K/AKT pathway significantly in TANs in the co-culture system by downregulating PI3K and p-AKT protein levels markedly (*P*<0.05), while there was no significant change in the total AKT. TGF-β/Smad signaling could significantly promote the expression of MMPs and other invasion and metastasis-related genes in tumor cells, thereby enhancing the ability of the cells to invade and metastasis 35. The activation of TGF-β/Smad signaling was also detected in our study. As shown in Figure 5D, 1D11 treatment significantly inhibited the level of TGF-β and SMAD7 in SW480 cells when tumor cells were co-cultured with TANs (*P*<0.05).

### TGF-β blockade delayed tumor growth *in vivo*

**Figure 6:**
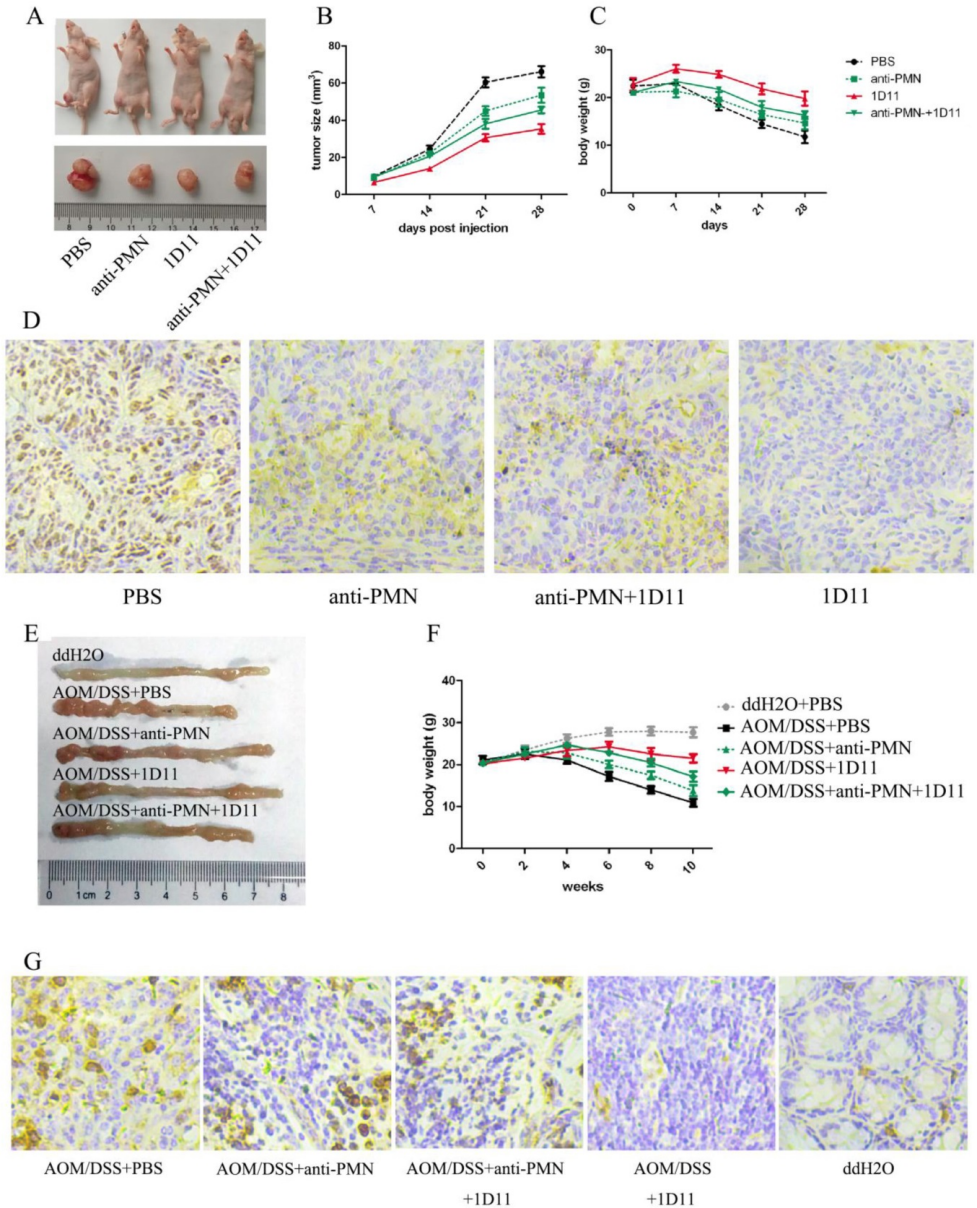
** TGF-β blockade delayed tumor growth *in vivo*.** The mice in xenograft model were divided into four group: PBS (xenograft mice only with PBS treatment), anti-PMN (PMN in the mice were eliminated with anti-Ly6G, three times per week, i.p, 5 mg/Kg), 1D11 (1D11 was used to inhibit the TGF-β expression in the mice, three times per week, i.p, 5 mg/Kg), and anti-PMN+1D11 (the mice were treated with anti-Ly6G and 1D11 simultaneously). The grouping methods used in the AOM/DSS model were same as those in xenograft model. In addition, the mice treated with ddH_2_O and PBS (ddH_2_O+PBS group) were set as negative controls for AOM/DSS model. A. Macroscopic observation of the tumor volumes in each group of mice in xenograft model. (We selected a mouse from each group as an example.) B. The tumor volumes in each group of mice in xenograft model. C. Body weights of mice in each group in xenograft model. D. The ki67 expression on tumor tissues in each group of mice in xenograft model. E. Macroscopic observation of the tumor formation in colons in each group of mice when the mouse was sacrificed. (We selected a mouse from each group as an example.) F. Body weights of mice in each group in AOM/DSS model. G. The expression of ki67 on tumor tissues in each group of mice in AOM/DSS model.

The anti-tumor effects of anti-TGF-β (1D11) on murine tumor model were explored in our study. As expected, the mice treated with 1D11 without PMN depletion obviously had smallest tumor volume compared with other groups (*P*<0.05), while PMN depletion weakened the anti-tumor effect of 1D11 (Figure 6A and Figure 6B). The body weight of each mouse was detected. Similarly, the mice treated with 1D11 without PMN depletion showed least body weight loss (Figure 6C, *P*<0.05), while there was a remarkable body weight loss in the other groups. At the same time, the proliferation ability of tumor cells was detected in the tumor tissues. As shown in Figure 6D, a lowest expression of ki67 was shown in the mice treated with 1D11 when compared with other groups (*P*<0.05). In addition, AOM/DSS-induced tumor model was used to confirm the anti-tumor effect of 1D11. As shown in Figure 6E and Figure 6F, 1D11 treatment without PMN depletion could significantly inhibit the formation of tumors and the body weight loss. Similarly, a lowest expression of ki67 was shown in the mice treated with 1D11 when compared with other mice (Figure 6G, *P*<0.05).

## Discussion

CRC remains a potentially lethal disease with a poor prognosis. This study is conducted to explore the effect of TGF-β inhibition on the polarization of TANs phenotype in CRC. TANs are considered to play a key role in tumor formation and angiogenesis 11,36. Numerous functional studies demonstrate that tumors could stimulate neutrophils to promote angiogenesis, migration and invasion of tumor cells. A markedly growing number of neutrophils also have been found in the submucosa and lamina propria of CRC patients, while PMN depletion reduces the infiltrated neutrophils and inhibits colon carcinomas progression 37, which suggests that neutrophils play an important role in CRC development. Targeted TANs in CRC may be a new therapeutic strategy for CRC. The present study shows that inhibition of TGF-β released by CRC tumor cells, on one hand, could induce the N2 phenotype of TANs polarization to N1 phenotype by increasing the cytotoxicity potential of TANs and decreasing the metastasis chemoattractants and immunosuppressive molecules produced by TANs; on the other hand, the inhibition could improve the expressions of GM-CSF and INF-γ in the tumor cells which promotes the activation of TANs. Ultimately, TGF-β blockade significantly improves the tumor cell apoptosis and inhibits the migration of tumor cells by suppressing the activation of the PI3K/AKT pathway in TANs and TGF-β/Smad signaling pathway in tumor cells. Furthermore, blocking TGF-β expression remarkably delays the tumor growth in CRC mouse model. Our data indicate that anti-TGF-β could polarize TANs to an anti-tumor phenotype and then inhibit the development of CRC. Our work also suggests that anti-TGF-β attenuated tumor growth is dependent on a synergistic combination of effects on both TANs and tumor cells in microenvironment. As far as we know, we demonstrate, for the first time, polarization of TANs towards an anti-tumor phenotype by inhibiting TGF-β expression could attenuate tumor growth in CRC.

In our study, high level of TGF-β was discovered in the tissue and serum of CRC patients. In addition, a high NLR ratio was found in the blood of CRC patients and an increasing number of neutrophils were found to be infiltrated into the CRC tumor tissues. Since tumor microenvironment could influence the phenotypes of TANs, whether blocking TGF-β in the tumor microenvironment could induce the N2 phenotype of TANs polarization to N1 phenotype? To answer this question, SW480 cells and primed neutrophils were co-cultured *in vitro* that simulated the TANs in the tumor microenvironment. The anti-TGF-β (1D11) which could neutralize the three isoforms of TGF-β was used to block the function of TGF-β in the tumor cells. As a result, inhibiting the level of TGF-β significantly increases the tumor cell apoptosis and inhibits tumor cell migration. It should be noted that, the most significant effect was presented in the group of SW480 and TANs co-culture system with 1D11 treated, but not the tumor cells treated with 1D11 only, which indicated that TANs have been polarized to an anti-tumor phenotype after treatment with 1D11 in the co-culture system. It is worth mentioning that, the apoptosis of TANs is also evaluated, and a lower apoptosis of TANs is shown in the co-cultured system when compared with the cells cultured alone. This finding could be consistent with the work by Andzinski et al that the life span of pro-tumor TANs is remarkably prolonged compared to the normal neutrophils 20. Our work indicates that the primed neutrophils co-cultured with SW480 tumor cells have acquired an N2 pro-tumor phenotype. After 1D11 treatment, the apoptosis of TANs appears to rise again, which indicates that the N2 phenotype of TANs is polarized to an anti-tumor phenotype gradually.

TANs could release several cytotoxic mediators such as NO, H_2_O_2_, and TNF-α. High level of their expression could lead to tumor cell apoptosis and display an anti-tumor phenotype. In this study, higher levels of NO, H_2_O_2_, and TNF-α were shown in TANs in the co-culture system treated with 1D11 when compared with other groups, which is consistent with Mishalian's 10 research, i.e. the anti-tumor TANs in two models of murine tumor, Lewis lung carcinoma and mesothelioma, showed more cytotoxic toward tumor cells with higher levels of TNF-α, NO and H_2_O_2_. Furthermore, the expression of ROS was detected in our work. ROS, the product of activated neutrophils, has been found to participate in tumor cell lysing and diminishing tumor cell proliferation. A higher level of ROS also had been found in TANs in the co-culture system after 1D11 treatment, which indicated that TGF-β inhibition could polarize TANs towards anti-tumor phenotype by increasing the potential of cytotoxic mediators in TANs in CRC.

Furthermore, 1D11 polarizes the anti-tumor phenotype of TANs by decreasing the metastasis chemoattractants in TANs. TANs acquire an N2 pro-tumor phenotype due to their high expressions of metastasis chemoattractants in the tumor microenvironment such as MMP9, IL-6, NE and low expression of ICAM-1, etc. Neutrophils are considered as the potent initiators of angiogenesis because they could release MMP-9 that contributes to tumorigenesis. IL-6, an inflammatory cytokine, promotes cell metastasis in tumor. ICAM-1, the adhesion molecule, is associated with neutrophil maturation and activation. NE plays an important role in promoting tumor cell proliferation. In our study, TGF-β blockade significantly decreases the mRNA and/or protein level of MMP9, IL-6, and NE, while increasing the level of ICAM-1 in TANs in the co-cultured system. Ultimately, the tumor cell metastasis is inhibited. Additionally, 1D11 polarizes the anti-tumor phenotype of TANs by decreasing the mediation of immunosuppression. TANs show a pro-tumor phenotype with promoting the mediation of immunosuppression 38. The high expression of Arg-1, an immune suppressive cytokine, may weaken the immune system of organism. To explore the immunity of TANs in the co-culture system after TGF-β blockading, arg-1 was detected in each group. In our study, a lower expression of arg-1 was shown in the co-culture system treated with 1D11. Previous studies had shown that TANs in untreated tumors produce matrix-degrading enzymes 39, accelerate the acquisition of a metastatic phenotype 40, and restrain the anti-tumor immune response 41. Our work supports the abovementioned findings and further demonstrates that TGF-β blockade could polarize the N2 pro-tumor phenotype of TANs towards N1 anti-tumor phenotype, which has a higher ability to destroy tumor cells in CRC.

High activity of neutrophils is considered as a characteristic of anti-tumor TANs since activated neutrophils could kill tumor cells and play a protective role for the host 38. GM-CSF and IFN-γ play an important role in neutrophil activation. GM-CSF could promote the growth and differentiate granulocyte lineages. It is produced by many cells of cancer including colon cancer 42,43. IFN-γ has been considered as a requisite factor in the tumor microenvironment for the differentiation of neutrophils 44. In our study, TGF-β blockade significantly increases the expressions of GM-CSF and IFN-γ in SW480 cells in the co-culture system, which promotes the activity of TANs. In summary, TGF-β blockade, on one hand, polarizes the N2 pro-tumor phenotype of TANs to N1 anti-tumor phenotype, and on the other hand increases the expression of GM-CSF and IFN-γ in the tumor cells, which promotes the activity of TANs.

The process of tumor progression is complex. One of the most studied mechanisms relates to PI3K/Akt signaling involvement. PI3K/Akt pathway plays an important role in the regulation of neutrophil chemotaxis. Activation of PI3K/Akt signaling has been shown to induce the production of pro-inflammatory cytokines and chemokine, and to potentially link to innate and adaptive immunity 13. In order to investigate whether PI3K/Akt signaling participates in the polarization of TANs, the related molecular mechanism was detected in our study. The results show that TGF-β blockade suppresses the activation of the PI3K/AKT pathway markedly by downregulating p-AKT and PI3K protein levels, while the total AKT has no significant change, which indicates TGF-β blocking inhibits PI3K/Akt-related pathway in TANs and then attenuates the chemoattractants secretion by neutrophils. Furthermore, TGF-β blockade significantly inhibits the level of TGF-β and SMAD7. TGF-β has been suggested to activate the Smad signaling pathway and significantly enhances invade and metastasis of tumor cells 42.These results suggest that the TGF-β blockade on CRC may be partly mediated by suppression of the PI3K/AKT signaling pathway in TANs and partly mediated by suppression of TGF-β/Smad signaling pathway in tumor cells.

Similarly, in the murine tumor model, the mice solely treated with 1D11 had smallest tumor volume compared with other groups. In addition, there was least body weight loss in the mice solely treated with 1D11. Besides, our study also reveals that the expression of ki67 is significantly inhibited after 1D11 treatment. A same result was also shown in the AOM/DSS-induced tumor model, which indicated that TGF-β blocking could significantly inhibit the development of CRC. It is particularly worth mentioning that the most significant inhibitory effect is shown in the mice solely treated with 1D11, but not the mice in anti-PMN+1D11 group, which indicates that the TANs in mice have been polarized to an anti-tumor phenotype and attenuate tumor growth in CRC.

In brief, TANs infiltration and high level of TGF-β in the tumor tissues play key role in CRC progression. In our study, blockage of TGF-β in tumor microenvironment could polarize the pro-tumor TANs towards an anti-tumor phenotype and ultimately suppresses human CRC *in vitro* and *in vivo*. In this sense, it serves as a new strategy for tumor treatment.

## Figures and Tables

**Table 1:** Baseline clinical characteristics of the enrolled patients

Clinic-pathologic parameters	Healthy volunteers (n %)	CRC (n %)
**Gender**		
Female	57(52)	53(49)
Male	53(48)	55(51)
**Age**	51(37-65)	53(42-67)
**Localization of primary tumor**		
Colon	-	74(69)
Rectum	-	34(31)
**Histology differentiation**		
Well	-	35(33)
Moderate	-	42(39)
Poor	-	31(28)
**Depth of invasion**		
T1-T2	-	49(45)
T3-T4	-	59(55)
**Lymph node metastasis**		
Present	-	45(42)
Absent	-	63(58)
**Ducks Stage**		
Ⅰ	-	34(31)
Ⅱ	-	31(29)
Ⅲ/Ⅳ	-	43(40)
**NLR**		
<3.0	94(85)	14(13)
>3.0	16(15)	94(87)
**Total**	110	108

**Table 2:** The sequences of real-time PCR primer

Gene name	Primer sequences (5'~3')
GAPDH F	GCGAGATCCCTCCAAAATC
GAPDH R	CATGAGTCCTTCCACGATACC
MMP9 F	TGGGTGGACCGGATGTTCCC
MMP9 R	GCCCACCTCCACTCCTCC
IL-6 F	CTTCGGTCCAGTTGCCTTCT
IL-6 R	GCCTCTTTGCTGCTTTCACAC
ICAM-1 F	CCTTCCTCACCGTGTACTGG
ICAM-1 R	AGCGTAGGGTAAGGTTCTTGC
ARG-1 F	AGGGTCCACCCTGATCTTGGAGT
ARG-1 R	GGAGAATCCTGGCACATCGGGA

## Abbreviations

TANs: tumor associated neutrophils
CRC: colorectal cancer
TGF-β: transforming growth factor β
UC: ulcerative colitis
NLR: neutrophil to lymphocyte ratio
NE: neutrophil elastase
MMP-2/9: matrix metalloproteinase 2/9
PMN: polymorphonuclear
Arg-1: arginase 1
FBS: fetal bovine serum
TNF-α: Tumor necrosis factor-α
GM-CSF: granulocyte-macrophage colony-stimulating factor
IFN-γ: interferon-γ
ROS: reactive oxygen species
AOM: Azoxymethane
DSS: dextran sulfate sodium
VEGF: Vascular endothelial growth factor
ELISA: Enzyme-linked immunosorbent assay
q-PCR: Quantitative real time PCR
